# Surgical Bunglers

**Published:** 1917-09

**Authors:** 


### Surgical Bunglers.

This is the title of an abstract of Dr. Arthur Dean Bevan's paper before the A. M. A., appearing in the Sun of N. Y. and repeated in the Buffalo Express and, doubtless, many other newspapers. The sub-title is also worth considering—"Too many operations by unskilled practitioners." A paper of this sort is proper and timely, just as proper and timely, for example, as one entitled "Neglect of Internal Medical Assistance by Operators," "Why is the Inexperienced General Practitioner Attempting Operations," "Where and How can the Man with Surgical Ambitions, Secure Adequate Clinical Training," "Better Doctoring for Less Money." There are all sorts of issues implying that the medical profession has not attained the ideal solution of a problem. The fact that such issues exist and proposed remedies ought to be put before the profession but there ought to be some practical method, an executive session, for instance, that would prevent their undue publicity. Such publicity leads to exaggerated notions of medical imperfections, hampers the influence of the profession in securing needed legislation even on matters entirely unassociated with the one discussed, gives a leverage to quacks of all kinds. There is also an ethical question involved which may best be illustrated by pointing out, with no intent of discourtesy, the immediate lay interpretation of the article mentioned. Put yourself in the position of the average lay reader of this particular article; you will infer from it, two things: 1. That Arthur Dean Bevan is a competent surgeon, a perfectly correct inference but one which ought not to be promulgated through the lay press; 2. That Drs. Smith, Jones, Robinson and others, one of whom is your family doctor and the others whom you know more or less well and favorably, socially or by reputation are at least subject to serious questions as to competency, an inference which may be correct in some cases but which is extremely unjust in others. When, as a physician, you have considered the effect of lay publicity to such articles, you will appreciate our contention for some adaptation of the principle of executive session, to all discussions that call into question the ability or integrity of the profession.

